# Supplementary material for: Real-time gastrointestinal infection surveillance through a cloud-based network of clinical laboratories
Source: PLoS One. 2021 Apr 30;16(4):e0250767. doi: 10.1371/journal.pone.0250767 (PMC8087049; doi:10.1371/journal.pone.0250767)
Supplement: Attachment — Submitted filename: Response to questions about funding statement.docx [file pone.0250767.s001.docx]

Juliana M. Ruzante
RTI International
3040 E. Cornwallis Rd PO Box 12194

Research Triangle Park, NC, 27709-2194 USA

March 10, 2021

Dear Editor,

Thank you once again for the opportunity to submit clarifications related to the manuscript “Real-time gastrointestinal infection surveillance through cloud-based network of clinical laboratories” (PONE-D-20-22634R1). We appreciate the opportunity to provide more information that can be used to update our financial and funding statements.

Below are our answers to the issues raised by the journal:

1. *Please provide contact information for the data owner*. Katherine Olin (Katherine.Olin@biofiredx.com)
2. *Please confirm that authors had no special access to the data, and that any qualified researcher can obtain access to the data in the same way the authors obtained it.* We confirm that. However, as mentioned in the response to reviewers, a data user agreement (DUA) is required to grant access to the data by a qualified researcher. That is part of the current terms and conditions that BioFire has with each facility participating in the Trend program.
3. *Thank you for providing the following information regarding your funding: "Biofire employees, Katherine Olin, Jeff Nawrocki, and Lindsay Meyers (who is no longer a BioFire employee), were part of the data analysis of this study and helped revising the manuscript. We revised the financial/funding statement to reflect that, please see below:“This study was funded by BioFire Diagnostics (*[*https://nam04.safelinks.protection.outlook.com/?url=https%3A%2F%2Fwww.biofiredx.com%2Fproducts%2Ffilmarray%2F&amp;data=04%7C01%7Cjruzante%40rti.org%7Ce089235adf7846c0e3da08d8e2796359%7C2ffc2ede4d4449948082487341fa43fb%7C0%7C0%7C637508358290331728%7CUnknown%7CTWFpbGZsb3d8eyJWIjoiMC4wLjAwMDAiLCJQIjoiV2luMzIiLCJBTiI6Ik1haWwiLCJXVCI6Mn0%3D%7C1000&amp;sdata=ErqXjBfRN%2BX0ZySyZWcPsUwNlNI%2FFkq6Gfn93rqQZOs%3D&amp;reserved=0*](https://nam04.safelinks.protection.outlook.com/?url=https%3A%2F%2Fwww.biofiredx.com%2Fproducts%2Ffilmarray%2F&amp;data=04%7C01%7Cjruzante%40rti.org%7Ce089235adf7846c0e3da08d8e2796359%7C2ffc2ede4d4449948082487341fa43fb%7C0%7C0%7C637508358290331728%7CUnknown%7CTWFpbGZsb3d8eyJWIjoiMC4wLjAwMDAiLCJQIjoiV2luMzIiLCJBTiI6Ik1haWwiLCJXVCI6Mn0%3D%7C1000&amp;sdata=ErqXjBfRN%2BX0ZySyZWcPsUwNlNI%2FFkq6Gfn93rqQZOs%3D&amp;reserved=0)*). Further, the authors: Katherine Olin, Jeff Nawrocki and Lindsay Meyers, under the direction of Dr. Ruzante, contributed to the data analysis and manuscript preparation. Katherine Olin and Jeff Nawrocki are paid employees of BioFire Diagnostics and Lindsay Meyers was a paid BioFire employee during the time of the study. The three of them hold stocks and shares of BioMérieux (owner of BioFire). Dr. Selvarangan is also on BioFire's Scientific Advisory Board, and has received funding for research from BioFire Diagnostics, as well as other companies.” Dr. Ruzante is current a co-PI in a proposal submitted to BioFire Diagnostics and the company paid for her travel expenses from San Diego to Salt Lake City in 2017 when Dr. Ruzante visited BioFire’s headquarter."*

*Please clarify the following:*

a. *Is the "funding for research from BioFire Diagnostics" received by Dr. Selvarangan related to the present study?* Dr. Selvarangan did not receive any funding for this study. His institution received grant funding for other BioFire studies in which he participated, not this one
b. *Please specify which companies are meant by the "other companies" that provided funding research to Dr. Selvarangan, and clarify whether any or all of these funding sources are related to the present study.* Dr. Selvarangan also received funding from Cepheid, Abbott, Becton and Dickinson, Hologic, Diasorin, Luminex, Merck and Bacterioscan. Nonetheless, none of those resources were used to conduct the present study.
c. *With regards to Dr. Ruzante, are either the "proposal submitted to BioFire Diagnostics" and/or "travel expenses from San Diego to Salt Lake City in 2017" related to the present study?* Dr. Ruzante is collaborating with researchers from Ohio State University (OSU) to conduct a new study that will be funded by BioFire. From the time the revisions were submitted to PLOS One to now, this new project was awarded to Ohio State and RTI will be a sub-awardee to OSU. Dr. Ruzante will serve as co-PI, but the new study is not related to the present one. As for the travel expenses in 2017, that trip was made to discuss, in general terms, opportunities for collaborations, and also for Dr. Ruzante to better understand the technology behind BioFire’s FilmArray diagnostic method and Trend.
4. *Are there any patents, products in development or marketed products associated with this research to declare?* There are no patents, products in development or marketed products associated with this research.

We greatly appreciate your help in updating our funding statement, so it is transparent and meet the guidelines of the journal.

Please address all correspondence concerning this manuscript to me at jruzante@rti.org.

Thank you for your consideration of this manuscript.

Sincerely,

Juliana M. Ruzante
